# Increasing prevalence of myopia and the impact of education in primary-school students in Xi'an, north-western of China

**DOI:** 10.3389/fpubh.2022.1070984

**Published:** 2022-12-19

**Authors:** Lu Ye, Yan-qi Yang, Guo-yun Zhang, Wen-jun Wang, Mei-xia Ren, Pan Ge, Jian Zhang, Nan Zhang, Xing-zhou Liu, Ming-lei Zhang, Yu-jiao Tong, Liang-cai Lu, Mo-qi Lv, Dang-xia Zhou, Cheng Pei

**Affiliations:** ^1^Health Science Center, Xi'an Jiaotong University, Xi'an, China; ^2^Shaanxi Eye Hospital, Xi'an People's Hospital (Xi'an Fourth Hospital), Xi'an, China; ^3^Department of Pathology, School of Basic Medical Sciences, Health Science Center, Xi'an Jiaotong University, Xi'an, China; ^4^Department of Ophthalmology, The First Affiliated Hospital of Xi'an Jiaotong University, Xi'an, China

**Keywords:** primary school, myopia, prevalence, cross-sectional studies, Xi'an city, north-western China

## Abstract

**Purpose:**

The present study was performed to detect the prevalence of myopia among primary-school students in Xi'an, north-western of China.

**Methods:**

The present study was a school-based study with students aged from 6 to 13 years old. All the individuals underwent ophthalmological examination and spherical equivalent (SE) of refractive error were measured with non-cycloplegic refraction. Myopia was defined as a SE of ≤ -0.5 diopters (D), and further divided into three stratified groups based on SE: low myopia (≤ -0.5 to >-3.0 D), moderate myopia (≤ -3.0 to >-6.0 D), and high myopia (≤ -6.0 D). Relative risk factors, including age, sex, grade and ethnicity were investigated using questionnaire.

**Results:**

A total of 4,680 individuals were eligible for this survey and 4,654 (99.4% participation rate) were finally included (51.2% boys). The mean age of participants was 8.756 ± 1.727 years. The whole city-level prevalence of total myopia was 57.1% (95% CI: 55.7–58.6%). Additionally, the prevalence of low, moderate, and high myopia was 45.0% (95% CI: 43.5–46.4%), 11.1% (95% CI: 10.2–12.0%), and 1.0% (95% CI: 0.7–1.3%), respectively. Moreover, grade (education level) instead of age, sex and ethnicity was the most essential risk factor for prevalence of overall myopia (OR = 1.844, 95% CI: 1.605–2.119), and an increase of prevalence by 84.4% per grade was seen. Furthermore, similar associations of grade were significant with low myopia (OR = 1.613, 95% CI: 1.385–1.877) and moderate myopia (OR = 2.186, 95% CI: 1.693–2.823), meanwhile, prevalence of low myopia and moderate myopia demonstrated an increase of prevalence by 61.3 and 118.6% per grade, respectively. None of the factors included in the present study was significant risk factor for high myopia.

**Conclusions:**

The present study investigated a non-negligible high prevalence of myopia among primary-school students in Xi'an, north-western of China, and a gradual increasing in proportion with education level.

## Introduction

Myopia (“near sightedness”) is reported as a major global health problem of twenty-first century (1), and is projected to affect approximate half of the world population by 2050 (2). Myopia is associated with various ocular diseases and contributes to a significant cause of vision loss (3). Myopia is also treated as an increasingly common refractive error among school-aged children worldwide (4), particularly in east Asia where around 80% of students completing middle school myopic (5–7).

In China, myopia prevention is an important public health priority, since the prevalence of myopia in Chinese school-aged students is one of the highest around the world (3, 8). Compared to western countries, this dramatic high prevalence of myopia may be attributable to Chinese specific cultures including early educational achievements, rigorous schooling system, frequent passing exams, and the long hours children spend studying, and the less time children spend outdoors (9, 10). Considering that children usually develop myopia at the age of 6 (2), prevention of myopia is needed in younger children, especially in primary-school students. Recently, the prevalence of myopia in primary-school students was reported in some large cities in south-eastern China (11–14), however, the prevalence of myopia among primary-school students in north-western China was scarcely reported.

Xi'an, which is the largest city in the north-western China, has more than 10 million population accounting for nearly one-third of the total population of Shaanxi Province (15). Compared with coastal and south-eastern regions of China, Xi'an has distinct geographic characteristics, cultural behaviors, and lifestyles, which have potential to affect the prevalence of myopia. Given this background, in order to detect the prevalence of myopia in Xi'an and to fill an important gap in prevalence of myopia around China, we performed this cross-sectional study to investigate the prevalence of myopia among primary-school students in Xi'an and to explore the potentially contributing factors to myopia.

## Materials and methods

### Study design and population

In 2021, the present study was performed to reveal the prevalence of myopia and its relative risk factors in primary-school students (from grades 1–6) in Xi'an, north-western of China. The sample size was calculated with a prevalence rate of 33.9% for myopia reported in previous studies (16) with a 2% error rate and a 95% confidence interval. Considering the non-response rate and the clustering design effect, which were assumed as 10 and 3%, respectively, the final ideal sample size was 956. All the students in this primary schools were eligible to participate in the study. Parent of each student was informed to sign a written consent forms before the ophthalmological examinations. This study followed the tenets of Declaration of Helsinki and was approved by the Institutional Medical Ethics Committee of Xi'an Jiaotong University.

### Ophthalmological examination and questionnaire

On the school premises, ophthalmological examinations were performed using the non-cycloplegic auto-refractometry (auto-refractor KR-800; Topcon Co., Tokyo, Japan). To assure data quality, the mean of three readings were taken. Spherical equivalent (SE) of the refractive error was calculated as the spherical refractive error plus half of the minus cylindrical refractive error. Based on the previous study (17), myopia was defined as a SE of ≤ -0.5 diopters (D) in the worse eye which had lower value of SE. Myopia was further divided into three categories: low myopia (SE: ≤ -0.5 to >-3.0 D), moderate myopia (SE: ≤ -3.0 to >-6.0 D), and high myopia (SE: ≤ -6.0 D) (8). The questionnaire items addressed potential risk factors including age, sex grade, and ethnicity.

### Statistical analysis

Continuous variables were presented as the mean ± standard deviation (SD), and categorical variables were presented as frequencies of the total. The prevalence of myopia was presented as a value and a 95% confidence interval (CI). Independent *t*-test was used to investigate differences between the two groups, and R by C chi-square test was used to analyze the differences of distribution from different groups. Spearman analysis was performed to investigate the association of SEs with age and grade.

Univariate logistic regression analyses were performed to examine potential associations between the prevalence of myopia with “myopia/non-myopia” as dependent variable and “relative risk factors including age, sex, grade, and ethnicity” as independent variable. Thereafter, multivariate logistic regression analyses with step-wise backward method were conducted to assess potential factors which were statistically significant in univariate analysis. Odds ratios (OR) and 95% confidence intervals (CIs) were presented in logistic regression analyses.

Stratified analyses were applied to determine whether various categories of myopia undertook different prevalence. Additionally, stratified univariate and multivariate logistic regression analyses were conducted to determine whether various categories of myopia undertook different risk factors for the development of myopia, using “low-myopia/non-low-myopia,” “moderate-myopia/non-moderate-myopia,” and “high-myopia/non-high-myopia” as dependent variable, independently. Furthermore, sensitivity analysis was performed to access the stability of our model.

All *P*-values were two-sided and *P* < 0.05 was considered to be statistically significant.

## Results

### Demographic characteristics

Four thousand six hundred and eighty children from public primary-school in south Xi'an were recruit in this study. Twenty-six individuals failed to finish the ophthalmological examination, 22 of which were absent on the examination day, one of which could not finish the examination due to the ocular trauma, and three of which rejected to perform the examination. Finally, 4,654 children were included in the present study with a mean age of 8.756 ± 1.727 years. Boys accounted for 51.2% (2,385/4,654) and Han ethnicity accounted for the majority (4,601/4,654, 98.9%). Among the included children, 17.4% students were in Grade 1, 17.2% students were in Grade 2, 17.3% students were in Grade 3, 17.0% students were in Grade 4, 16.4% students were in Grade 5, and 14.7% students were in Grade 6. The details of demographic data were shown in Table 1.

**Table 1:** Demographic factors associated with myopia in children.

**Variables**	**Total**	**Myopia**	**Non-myopia**	***P*-value**
Total, *n* (%)	4,654	2,659 (57.1)	1,995 (42.9)	
Age (year, mean ± SD)	8.756 ± 1.727	9.351 ± 1.569	7.961 ± 1.598	< 0.001
**Sex**, ***n*** **(%)**
Boys	2,385	1,348 (56.5)	1,037 (43.5)	0.386
Girls	2,269	1,311 (57.8)	958 (42.2)	
Grade, *n* (%)
1	811	162 (20.0)	649 (80.0)	< 0.001
2	799	325 (40.7)	474 (59.3)	
3	806	493 (61.2)	313 (38.8)	
4	790	522 (66.1)	268 (33.9)	
5	763	608 (79.7)	155 (20.3)	
6	685	549 (80.1)	136 (19.9)	
Ethnicity, *n* (%)
Han	4,601	2,627 (57.1)	1,974 (42.9)	0.631
Other ethnic groups	53	32 (60.4)	21 (39.6)	

### Spherical equivalent of refractive errors among ages, sex, grades, and ethnicity

**Figure 1:**
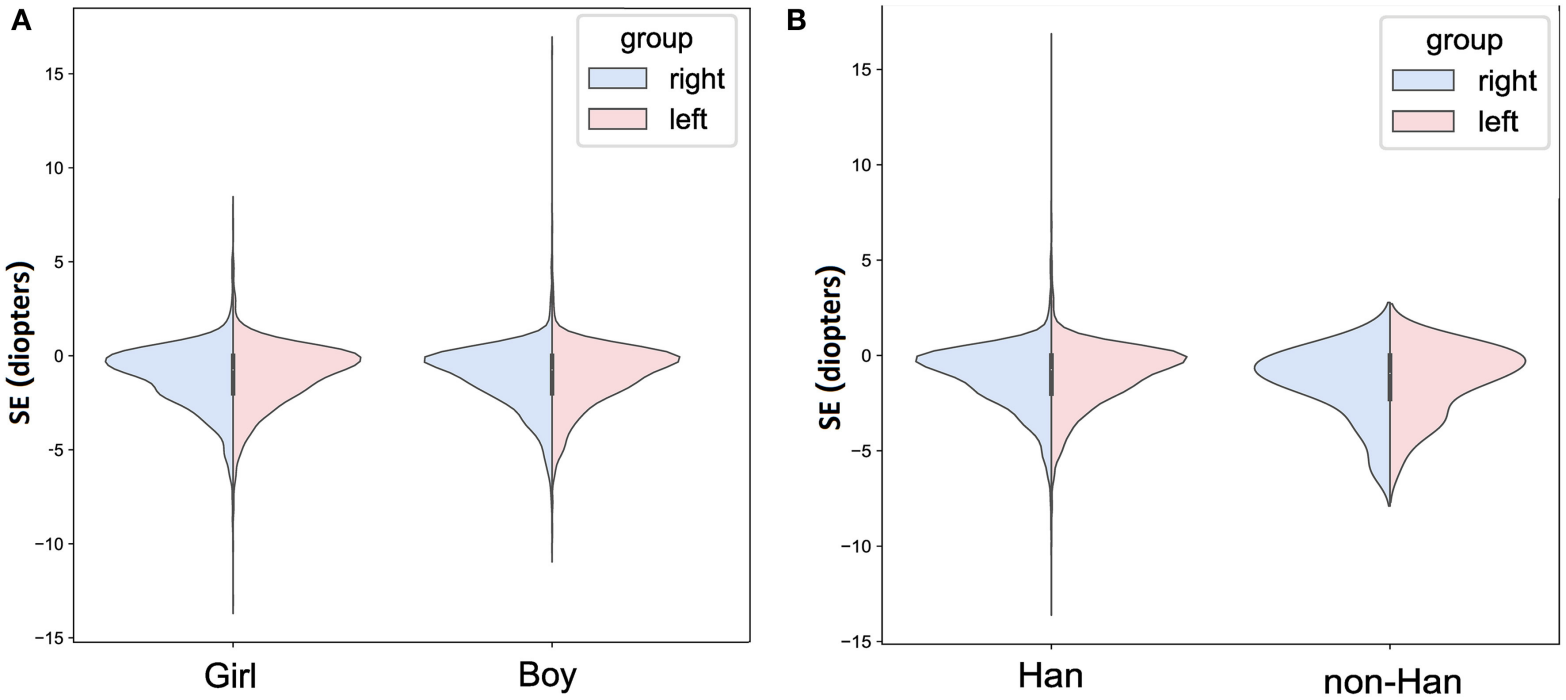
The bilateral spherical equivalent in different age or grade in primary-school students in Xi'an. **(A)** The bilateral spherical equivalent in different age; **(B)** the bilateral spherical equivalent in different grade. SE, spherical equivalent.

**Figure 2:**
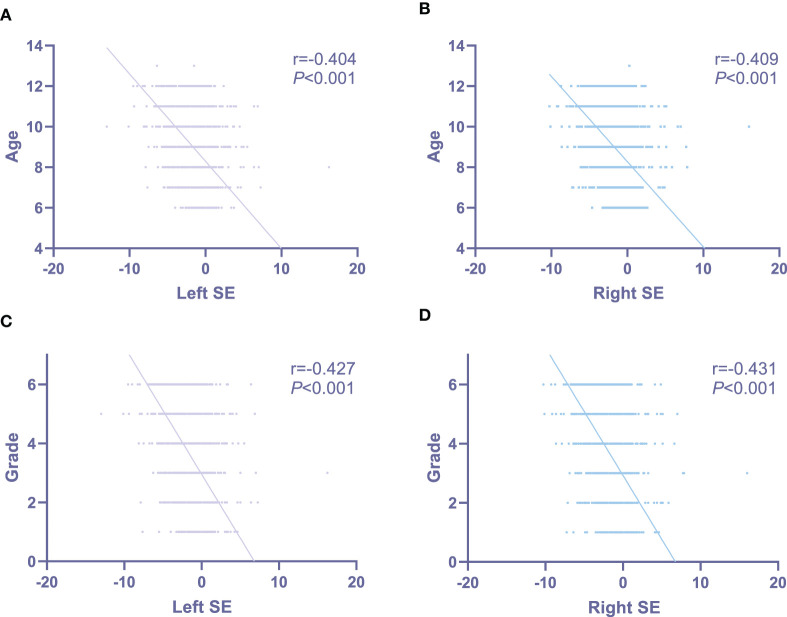
The associations of bilateral spherical equivalent with age and grade in primary-school students in Xi'an. **(A)** The associations of left spherical equivalent with age; **(B)** the associations of right spherical equivalent with age; **(C)** the associations of left spherical equivalent with grade; **(D)** the associations of right spherical equivalent with grade. SE, spherical equivalent.

Mean SE for the left and right eyes were −1.070 ± 1.664 D and −1.138 ± 1.680 D. In right eye, no difference of SE was observed between girls and boys (girls: −1.125 ± 1.667 D, boys: −1.151 ± 1.693 D; *P* = 0.597; Figure 1A), as well as between Han and non-Han children (Han ethnicity: −1.135 ± 1.678 D, non-Han ethnicity: −1.459 ± 1.821 D; *P* = 0.163; Figure 1B). In addition, SE was negatively associated with age (r for Pearson = −0.409, *P* < 0.001; Figure 2B) and grade (*r* for Pearson = −0.431, *P* < 0.001; Figure 2D).

Similar results were seen in left eye (Figures 1, 2).

### Prevalence of myopia

#### Overall analysis

**Figure 3:**
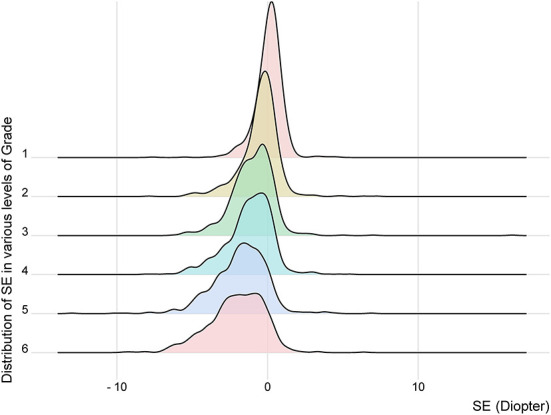
The distribution of spherical equivalent in various levels of grade. SE, spherical equivalent.

The overall prevalence of myopia in primary-school students in Xi'an was 57.1% (95% CI: 55.7–58.6%; Table 2). The prevalence of myopia increased signifcantly with grades (*P* < 0.001, Table 1). It was 20.0% (95% CI: 17.2–22.7%), 40.1% (95% CI: 37.3–44.1%), 61.2% (95% CI: 57.8–64.5%), 66.1% (95% CI: 62.8–69.4%), 79.7% (95% CI: 76.8–82.5%), and 80.1% (95% CI: 77.2–83.1%) in Grade 1, 2, 3, 4, 5, and 6, respectively (Tables 1, 2; Figure 3). No statistically significant difference in prevalence of myopia was seen between boys and girls (*P* = 0.159, Table 1), as well as between Han and non-Han ethnicity (*P* = 0.631, Table 1).

**Table 2:** Prevalence rate and categories of myopia stratified by age, sex, grade, and ethnicity.

**Variables**	**Myopia (SE:** ≤ **-0.5 D)**		**Myopia categories**						
			**Low (SE:** ≤ **-0.5 to** >**-3.0 D)**		**Moderate (SE:** ≤ **-3.0 to** >**-6.0 D)**		**High (SE:** ≤ **-6.0 D)**		
	***n*** **(%)**	**95% CI**	***n*** **(%)**	**95% CI**	***n*** **(%)**	**95% CI**	***n*** **(%)**	**95% CI**	
Total	2,659 (57.1)	55.7–58.6	2,093 (45.0)	43.5–46.4	518 (11.1)	10.2–12.0	48 (1.0)	0.7–1.3	
**Sex**									
Boys	1,348 (56.5)	54.5–58.5	1,043 (43.7)	41.7–45.7	282 (11.8)	10.5–13.1	23 (1.0)	0.6–1.4	χ^2^ = 3.678; *P* = 0.159
Girls	1,311 (57.8)	55.7–59.8	1,050 (46.3)	44.2–48.3	236 (10.4)	9.1–11.7	25 (1.1)	0.7–1.5	
**Grade**									
1	162 (20.0)	17.2–22.7	155 (19.1)	16.4–21.8	5 (0.6)	0.1–1.2	2 (0.2)	−0.1 to 0.6	χ^2^ = 141.708; *P* < 0.001
2	325 (40.1)	37.3–44.1	292 (36.6)	33.2–39.9	32 (4.0)	2.6–5.4	1 (0.1)	−0.1 to 0.4	
3	493 (61.2)	57.8–64.5	431 (53.5)	50.0–56.9	60 (7.4)	5.6–9.3	2 (0.5)	−0.1 to 0.6	
4	522 (66.1)	62.8–69.4	412 (52.2)	48.7–55.6	104 (13.2)	10.8–15.5	6 (0.8)	0.2–1.4	
5	608 (79.7)	76.8–82.5	429 (56.2)	52.7–59.7	161 (21.1)	18.2–24.0	18 (2.4)	1.3–3.4	
6	549 (80.1)	77.2–83.1	374 (54.6)	50.9–58.3	156 (22.8)	19.6–25.9	19 (2.8)	1.5–4.0	
**Ethnicity**									
Han	2,627 (57.1)	55.7–58.5	2,070 (45.0)	43.6–46.4	510 (11.1)	10.2–12.0	47 (1.0)	0.7–1.3	χ^2^ = 1.011; *P* = 0.603
Non-han	32 (60.4)	47.2–73.5	23 (43.4)	30.1–56.7	8 (15.1)	5.5–24.7	1 (1.9)	−1.8 to 5.5	
Age (mean ± SD)	9.351 ± 1.569		9.179 ± 1.587^*, #^		9.958 ± 1.320		10.333 ± 1.277		

^*^Compared with moderate myopia.

^#^Compared with high myopia.

SE, spherical equivalent; d, diopters.

#### Stratified analyses

**Figure 4:**
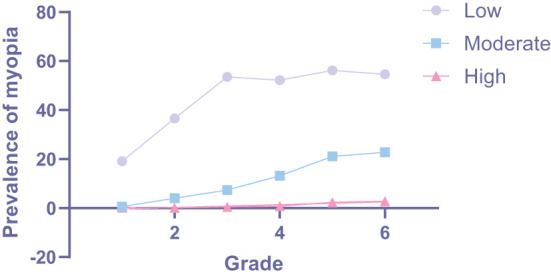
Grade specific incidence of in different categories of myopia.

When stratified according to myopia categories, low myopia showed the highest prevalence (45.0%, 95% CI: 43.5–46.4%), followed by moderate (11.1%, 95% CI: 10.2–12.0%), and high myopia (1.0%, 95% CI: 0.7–1.3%, Table 2). Additionally, children with low myopia had significant lower age compared with individuals with moderate (*P* < 0.001, Table 2) and high myopia (*P* < 0.001, Table 2). Moreover, grades could alter distributions of prevalence in different myopia categories (χ^2^ = 141.708; *P* < 0.001). Low myopia prevalence significantly increased with increasing grade, ranging from 19.1% in grade 1–54.6% in grade 6 with a slope of 6.714 (*P* = 0.031, Figure 4). Likewise, similar increasing prevalence with more gentle slopes were showed in moderate and high myopia (Table 2; Figure 4). Furthermore, sex (χ^2^ = 3.678; *P* = 0.159) and ethnicity (χ^2^ = 1.011; *P* = 0.603) could not alter the distributions of prevalence in different myopia categories (Table 2).

### Risk factors for the development of myopia

#### Overall analysis

Univariate logistic regression analyses were employed to assess the association between myopia and relevant factors including age, sex grade, and ethnicity. In the present study, higher prevalence of myopia was associated with older age (OR = 1.696, 95% CI: 1.629–1.766; *P* < 0.001; Table 3) and higher grade (OR = 1.762, 95% CI: 1.690–1.837; *P* < 0.001; Table 3). However, no significant associations of the risk of myopia was observed with sex (OR = 0.950, 95% CI: 0.846–1.067; *P* = 0.386; Table 3) and ethnicity (OR = 0.873, 95% CI: 0.502–1.519; *P* = 0.632; Table 3).

**Table 3:** Associations between the prevalence of myopia and associated factors.

**Variables**	**Univariate logistic regression analysis**			**Multivariate logistic regression analysis**		
	**OR**	**95% CI**	***P-*value**	**OR**	**95% CI**	***P-*value**
Age (year)	1.696	1.629–1.766	< 0.001	0.955	0.834–1.092	0.5
Sex (boys vs. girls)	0.950	0.846–1.067	0.386			
Grade	1.762	1.690–1.837	< 0.001	1.844	1.605–2.119	< 0.001
Ethnicity (han vs. non-han)	0.873	0.502–1.519	0.632			

OR, odds ratio; CI, confidence interval.

Moreover, in order to further analyze the factors contributing to the prevalence of myopia, multivariate logistic regression analysis was performed on age and grade, all of which had demonstrated a notable association with the prevalence of myopia in the univariate logistic regression analysis. Consequently, we found that only the higher grade (OR = 1.844, 95% CI: 1.605–2.119; *P* < 0.001; Table 3) continued to show a significant association with higher prevalence of myopia. Specifically, an increase in grade showed significant association with a 84.4% increase in the relative risk of myopia. In that model, prevalence of myopia was no longer significantly associated with age (OR = 0.955, 95% CI: 0.834–1.092; *P* = 0.500; Table 3).

#### Stratified analyses

When stratified according to myopia categories, univariate logistic regression analysis indicated that high prevalence of low myopia remained significantly associated with older age (OR = 1.563, 95% CI: 1.495–1.7633; *P* < 0.001; Table 4) and higher grade (OR = 1.612, 95% CI: 1.540–1.687; *P* < 0.001; Table 4). And multivariate logistic regression analysis demonstrated that only the higher grade (OR = 1.613, 95% CI: 1.385–1.877; *P* < 0.001; Table 4) continued to show a significant association with higher prevalence of low myopia. Specifically, an increase in grade showed significant association with a 61.3% increase in the relative risk of low myopia.

**Table 4:** Associations between the prevalence of different categories of myopia and associated factors.

**Variables**	**Univariate analysis**			**Multivariate analysis**		
	**OR**	**95% CI**	***P-*value**	**OR**	**95% CI**	***P-*value**
**Low**^**#1**^ **(SE:** **≤-0.5 to** **>-3.0 D)**						
Age (year)	1.563	1.495–1.633	< 0.001	1.001	0.863–1.158	0.996
Sex (boys vs. girls)	0.886	0.778–1.009	0.069			
Grade	1.612	1.540–1.687	< 0.001	1.613	1.385–1.877	< 0.001
Ethnicity (han vs. non-han)	1.029	0.553–1.913	0.929			
**Moderate**^**#2**^ **(SE:** **≤-3.0 to** **>-6.0 D)**						
Age (year)	2.137	1.980–2.307	< 0.001	1.028	0.803–1.314	0.828
Sex (boys vs. girls)	1.066	0.874–1.300	0.53			
Grade	2.246	2.074–2.432	< 0.001	2.186	1.693–2.823	< 0.001
Ethnicity (han vs. non-han)	0.729	0.315–1.686	0.459			
**High**^**#3**^ **(SE:** **≤-6.0 D)**						
Age (year)	2.353	1.915–2.892	< 0.001	1.358	0.741–2.488	0.322
Sex (boys vs. girls)	0.821	0.462–1.458	0.5			
Grade	2.513	2.001–3.157	< 0.001	1.856	0.978–3.520	0.058
Ethnicity (han vs. non-han)	0.537	0.070–4.108	0.549			

OR, odds ratio; CI, confidence interval; SE, spherical equivalent; D, diopters.

#1: this model was performed with “low-myopia (SE: ≤ -0.5 to >-3.0 D)” and “non-low-myopia (SE: >-0.5 and ≤ -3.0 D)” as dependent variable.

#2: this model was performed with “moderate-myopia (SE: ≤ -3.0 to >-6.0 D)” and “non-moderate-myopia (SE: >-3.0 and ≤ -6.0 D)” as dependent variable.

#3: this model was performed with “high-myopia (SE: ≤ -6.0 D)” and “non-high-myopia (SE: >-6.0 D)” as dependent variable.

Similarity, in moderate myopia group, univariate logistic regression analysis showed significant association of the high prevalence with older age (OR = 2.137, 95% CI: 1.980–2.307; *P* < 0.001; Table 4) and higher grade (OR = 2.246, 95% CI: 2.074–2.432; *P* < 0.001; Table 4). And multivariate logistic regression analysis confirmed the statistic association only with higher grade (OR = 2.186, 95% CI: 1.693–2.823; *P* < 0.001; Table 4). Specifically, an increase in grade showed significant association with a 118.6% increase in the relative risk of moderate myopia.

However, in high myopia group, although age (OR = 2.353, 95% CI: 1.915–2.892; *P* < 0.001; Table 4) and grade (OR = 2.513, 95% CI: 2.001–3.157; *P* < 0.001; Table 4) indicated positive associations with prevalence of myopia in univariate logistic regression analysis, the two factors failed to be verified as significant relative risk factors for myopia in multivariate logistic regression analysis (age: OR = 1.358, 95% CI: 0.741–2.488; *P* = 0.322; grade: OR = 1.856, 95% CI: 0.978–3.520; *P* = 0.058; Table 4).

### Sensitivity analyses

Associations between prevalence of myopia and relative risk factors in girls, in boys, and in Han individuals were generally similar to those in the overall group, respectively (Table 5).

**Table 5:** Detail of sensitivity analyses of logistic regression models in all samples.

		**Variables**	**OR**	**95% CI**	***P-*value**
Sex	Girls	Age (year)	1.7	1.604–1.802	< 0.001
		Grade	1.775	1.671–1.885	< 0.001
		Ethnicity (han vs. non-han)	0.722	0.320–1.626	0.432
	Boys	Age (year)	1.698	1.606–1.796	< 0.001
		Grade	1.755	1.657–1.859	< 0.001
		Ethnicity (han vs. non-han)	1.04	0.485–2.232	0.919
Excluded non-Han children		Age (year)	1.696	1.629–1.767	< 0.001
		Sex (boys vs. girls)	0.954	0.849–1.072	0.427
		Grade	1.765	1.692–1.840	< 0.001

OR, odds ratio; CI, confidence interval.

## Discussion

### Our findings

To our knowledge, the present study is the first school-based study to assess the prevalence of myopia in primary-school students in Xi'an, north-western of China. The present study indicated four key findings: (1) the total prevalence of myopia in students from public primary-school in Xi'an, north-western China was 57.1%; (2) the prevalence of low, moderate, and high myopia was 45.0, 11.1, and 1.0%, respectively; (3) grade instead of age, sex, and ethnicity was the most essential risk factor for prevalence of myopia, specifically an increase in grade indicated significant association with a 84.4% increase in the relative risk of myopia; (4) similar associations of grade were seen with low and moderate myopia, and prevalence of low and moderate myopia demonstrated a increase by 61.3 and 118.6% per grade, respectively; however, none of the factors included in the present study was significant risk factor for high myopia.

### Compared to previous studies

**Figure 5:**
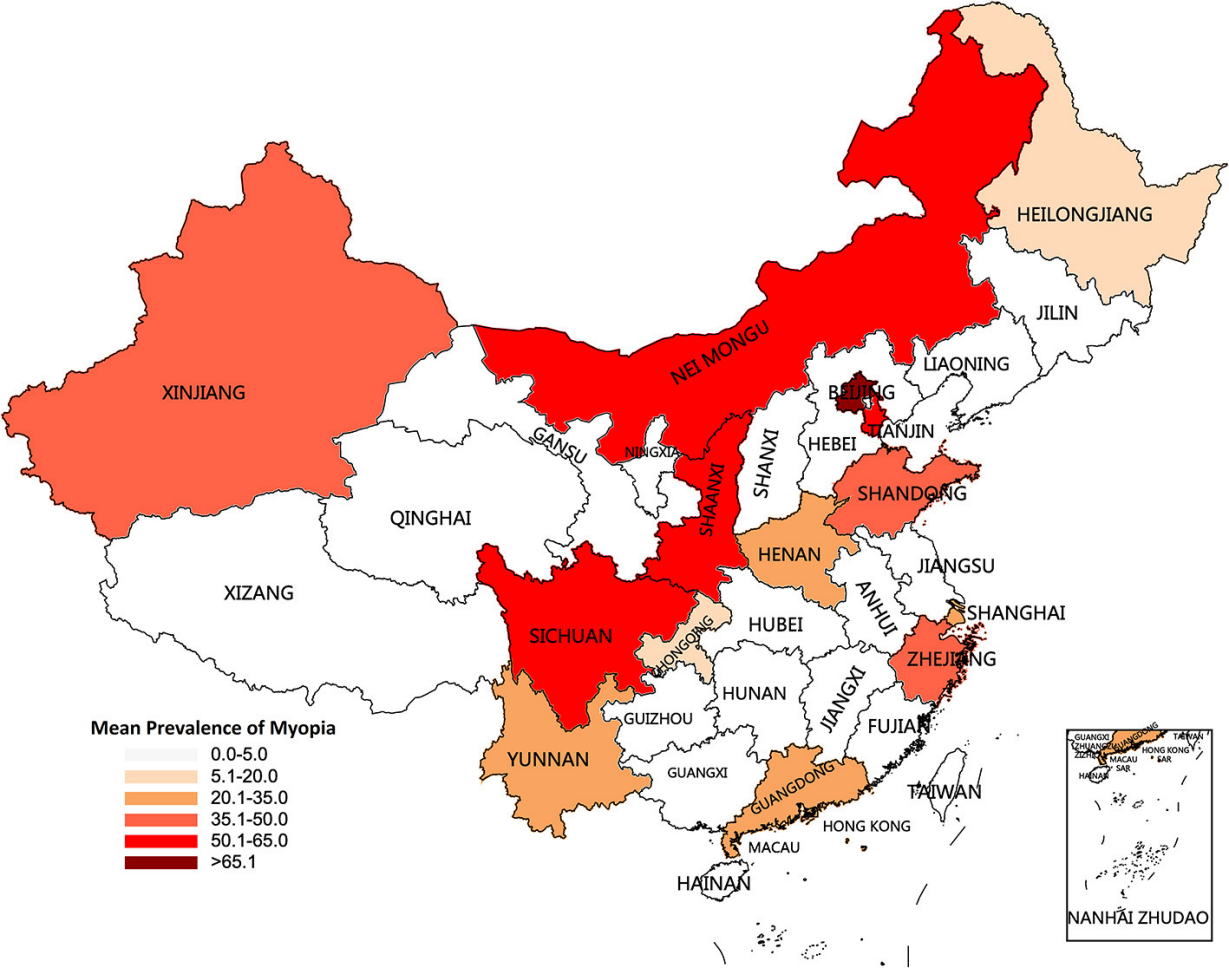
Characteristics and mean prevalence of myopia for the recent decade (1998–2021) by province in the current study, Dong et al. (18), Pan et al. (19), Shi et al. (20), Liu et al. (21), Yam et al. (12), Thorn et al. (13), and Xu et al. (22).

Several previous researches reported the prevalence of myopia among Chinese children and adolescent strongly suggesting a non-negligible health issue in ophthalmology, and triggered studies into the reason for the high prevalence, aiming for prevention. As shown in Table 6 and Figure 5, Dong et al. (18) summarized 19 population-based studies selecting data from 1998 to 2016 around the whole China and indicted that the pooled prevalence of myopia was 37.7% in children with 3–19 years old. This lower prevalence might result from the highly increased prevalence (2) and the different cycloplegia refraction (23–25). In addition, Pan et al. (19) and Shi et al. (20) reported that the prevalence of myopia was 29.5% and 47.5 in south-western China and western China, respectively. Western and south-western China have more ethnic diversity and less study burden which were confirmed to be relative factors for the development of myopia (26, 27). This might be essential reasons for the differences between the studies of Pan et al./Shi et al. and ours.

**Table 6:** The prevalence of myopia around China by published studies.

**References**	**Year of data collection**	**City**	**Country STUDY design**	**Sample size (*N*)**	**Age(grade)**	**Cycloplegic refraction**	**Myopia definition**	**Prevalence (%)**
The present study	2021	Xi'an	North-western	4,654	6–13 (1–6)	Non-cycloplegic refraction	≤ -0.5 D	57.1
Dong et al. (18)	1998–2016	Muti-city	Around China	192,569	3–19	Cycloplegic refraction	≤ -0.5 D	37.7
Pan et al. (19)	2016	Mo Jiang	South-western	2,346	13–14	Cycloplegic refraction	≤ -0.5 D	29.5
Shi et al. (20)	2019	Urumqi	North-western	6,883	7–20	–	≤ -0.5 D	47.5
Liu et al. (21)	2016	Tian Jin	North-eastern	566	6–14	Cycloplegic refraction	≤ -0.5 D	59.2
Yam et al. (12)	–	Hong Kang	South-eastern	4,257	6–8	Non-cycloplegic refraction	≤ -0.5 D	25.0
Thorn et al. (13)	–	Wen Zhou	South-eastern	13,220	(1–6)	Non-cycloplegic refraction	≤ -1.0 D	49.5
Xu et al. (22)	2019	Wen Zhou	South-eastern	580,609	(1–6)	Non-cycloplegic refraction	≤ -1.0 D	38.2

D, diopters.

As for primary-school students, Liu et al. (21) revealed a prevalence of 59.2% among children in Tianjin, which was in accord with our findings. Yam et al. (12) analyzed data from children with 6–8 years old and found the prevalence was 25.0%. The lower prevalence might due to the younger age and less study burden (27). Thorn et al. (13) and Xu et al. (22) claimed that the prevalence of myopia was 49.5 and 38.1% in south-eastern China, respectively, which was lower than that in our study. The standard in the two studies was defined myopia as SE ≤ −1.0 D, which was stricter than ours. This was one of the reasons for these differences. Some other underlying reasons might be contributed to geographic differences implying variations of ethnicity (26), socio-economic status (28), life/study style (29–31), air condition (1), educational pressures (32), and genetic background (33).

### The possible factors influencing development of myopia

Apart from age, ethnicity, educational pressures, socio-economic status, study style, air condition, and genetic background mentioned upon, sex and grade (education level) were some other most well-known factors affecting development of myopia.

The correlation between sex and the prevalence of myopia remained confused. Majority studies revealed that girls had higher prevalence of myopia when compared with boys (13, 27, 34–36), however, our findings show little difference of the prevalence of myopia between girls and boys. The discrepancy might be result from the different distribution of age and the different definition of myopia.

A cross-sectional study enrolled 812,979 youths across five surveys in China by Zhang et al. (37) demonstrated that grade or education level was associated with a decrease in mean spherical equivalent which was equal to an increase in the prevalence of myopia, which was consistent with our conclusion. Similar results were demonstrated by other researches in Europe and the U.S. (38–40). Preparing for examinations, pressure and competition, and longer digital screen time stemming from higher grade will enhance spherical equivalent and increase the prevalence of myopia. Noteworthily, popularization of digital screen has been cited as a potential modifiable environmental risk factor that can increase myopia risk (41, 42). More smartphones, pads and telescreens used in children's daily life and study could partly explain the increasing prevalence and the younger-age trend of myopia.

### Myopia categories

As it wildly known, high myopia had potential to cause vision-threatening complications, including choroid neovascularization, rhegmatogenous retinal detachment, and macular hemorrhage, which harms public visual health (43, 44). Thus, preventing occurrence and progression of high myopia is an essential public health issue. Our findings indicated that low and moderate myopia showed shaper trends of increase in prevalence with grade, however, high myopia showed an extremely gentle increase in prevalence with grade. Likewise, grade was confirmed to be a risk factor for low and moderate myopia, but not for high myopia. Additionally, age, sex, and ethnicity were also failed to be verified as relative factors for high myopia. This was different from previous studies, which revealed a significant association between high myopia and age (45, 46). One of the possible explanations was the various distribution of grade. Previous studies recruited students covering primary-school, middle-school, and high-school, however, our study only included students from primary-school. Compared with individuals in primary and middle school, high school students particularly exhibited prominent higher prevalence of high myopia (27). The small sample size might bias our analyses. Another possible explanation was that, since myopia might be a result of gene-environment interactions, younger children with low and moderate myopia was more inclined to attributing to environment factor, however, younger children with high myopia was more inclined to attributing to genetic background (47).

### Limitations

This study has several limitations. First, considering that this was a retrospective study, the possibility of observation and inclusion biases could not be rules out. Although we have conducted sensitivity analyses to confirm our statistical models were stable and robust, further studies with larger sample sizes were expected to relieve this bias. Second, non-cycloplegia refraction was conducted attribute to the large-scale investigation, which would overestimate the myopia prevalence of children. However, this might not pose serious measurement errors for the present study since it was the annual shift that was of interest instead of absolute values. Thirdly, we mainly assessed the associations between prevalence of myopia and some potential risk factors including age, sex grade, and ethnicity. Considering the limited understanding ability of primary-school students, we simplified the questionnaire. Hence, some certain other related factors, such as daily eye habits, daily study habits, and lifestyle habits were not collected in the present study. More comprehensive myopia-related factors collected with the help of parents are expectant to be applied in further studies.

## Conclusions

In summary, the present study was the first study to reveal a non-negligible high prevalence of myopia in primary-school students in Xi'an, north-western of China. Higher prevalence of myopia was significantly associated with an increasing level of education (grade). Our findings indicated serious reproductive health warnings and appealed to more attention to preventing the progression of myopia.

## Data availability statement

The raw data supporting the conclusions of this article will be made available by the authors, without undue reservation.

## Ethics statement

The studies involving human participants were reviewed and approved by the Institutional Medical Ethics Committee of Xi'an Jiaotong University. Written informed consent to participate in this study was provided by the participants' legal guardian/next of kin.

## Author contributions

CP, M-qL, and D-xZ: conception and design of the study. LY, G-yZ, W-jW, M-xR, X-zL, M-lZ, Y-jT, and L-cL: data collection. Y-qY and PG: illustrations, statistical analysis, and interpretation of data. LY, CP, JZ, and NZ: administrative, technical, or material support. M-qL and LY: drafting of the manuscript. D-xZ and M-qL: critical revision of the manuscript. JZ and CP: study supervision. All authors read and approved the final manuscript.

## References

[1] Pan CW Ramamurthy D Saw SM. Worldwide prevalence and risk factors for myopia. Ophthal Physiol Optics. (2012) 32:3–16. 10.1111/j.1475-1313.2011.00884.x 22150586

[2] Holden BA Fricke TR Wilson DA Jong M Naidoo KS Sankaridurg P. Global prevalence of myopia and high myopia and temporal trends from 2000 through 2050. Ophthalmology. (2016) 123:1036–42. 10.1016/j.ophtha.2016.01.006 26875007

[3] Wang J Ying GS Fu X Zhang R Meng J Gu F. Prevalence of myopia and vision impairment in school students in Eastern China. BMC Ophthalmol. (2020) 20:2. 10.1186/s12886-019-1281-0 31898504 PMC6941318

[4] Morgan IG Ohno-Matsui K Saw SM. Myopia. Lancet. (2012) 379:1739–48. 10.1016/S0140-6736(12)60272-4 22559900

[5] Lin LL Shih YF Hsiao CK Chen CJ. Prevalence of myopia in Taiwanese schoolchildren: 1983 to 2000. Ann Acad Med Singapore. (2004) 33:27–33. 15008558

[6] Wu HM Seet B Yap EP Saw SM Lim TH Chia KS. Does education explain ethnic differences in myopia prevalence? A population-based study of young adult males in Singapore. Optomet Vis Sci. (2001) 78:234–9. 10.1097/00006324-200104000-00012 11349931

[7] You QS Wu LJ Duan JL Luo YX Liu LJ Li X. Prevalence of myopia in school children in greater Beijing: the Beijing Childhood Eye Study. Acta Ophthalmol. (2014) 92:e398–406. 10.1111/aos.12299 25165786

[8] Guo L Yang J Mai J Du X Guo Y Li P. Prevalence and associated factors of myopia among primary and middle school-aged students: a school-based study in Guangzhou. Eye. (2016) 30:796–804. 10.1038/eye.2016.39 26965016 PMC4906452

[9] Zhao J Pan X Sui R Munoz SR Sperduto RD Ellwein LB. Refractive error study in children: results from Shunyi District, China. Am J Ophthalmol. (2000) 129:427–35. 10.1016/S0002-9394(99)00452-3 10764849

[10] Li L Zhong H Li J Li CR Pan CW. Incidence of myopia and biometric characteristics of premyopic eyes among Chinese children and adolescents. BMC Ophthalmol. (2018) 18:178. 10.1186/s12886-018-0836-9 30029645 PMC6053817

[11] Ma Y Qu X Zhu X Xu X Zhu J Sankaridurg P. Age-specific prevalence of visual impairment and refractive error in children aged 3-10 years in Shanghai, China. Invest Ophthalmol Vis Sci. (2016) 57:6188–96. 10.1167/iovs.16-20243 27842160

[12] Yam JC Tang SM Kam KW Chen LJ Yu M Law AK. High prevalence of myopia in children and their parents in Hong Kong Chinese population: the Hong Kong Children Eye Study. Acta Ophthalmol. (2020). 10.1111/aos.14350 31981300

[13] Thorn F Chen J Li C Jiang D Chen W Lin Y. Refractive status and prevalence of myopia among Chinese primary school students. Clin Exp Optomet. (2020) 103:177–83. 10.1111/cxo.12980 31674055

[14] Lam CS Lam CH Cheng SC Chan LY. Prevalence of myopia among Hong Kong Chinese schoolchildren: changes over two decades. Ophthal Physiol Optics. (2012) 32:17–24. 10.1111/j.1475-1313.2011.00886.x 22150587

[15] Wang JY Zhang KG Ruan JX Chen W Wang L. Shift in HIV/AIDS epidemic and factors associated with false positives for hiv testing: a retrospective study from 2013 to 2018 in Xi'an, China. Curr HIV Res. (2020) 18:219–26. 10.2174/1570162X18666200415123607 32294041 PMC7475938

[16] Xie Z Long Y Wang J Li Q Zhang Q. Prevalence of myopia and associated risk factors among primary students in Chongqing: multilevel modeling. BMC Ophthalmol. (2020) 20:146. 10.1186/s12886-020-01410-3 32295555 PMC7161106

[17] Hashemi H Rezvan F Ostadimoghaddam H Abdollahi M Hashemi M Khabazkhoob M. High prevalence of refractive errors in a rural population: 'Nooravaran Salamat' Mobile Eye Clinic experience. Clin Experiment Ophthalmol. (2013) 41:635–43. 10.1111/ceo.12071 23331326

[18] Dong L Kang YK Li Y Wei WB Jonas JB. Prevalence and time trends of myopia in children and adolescents in China: a systemic review and meta-analysis. Retina. (2020) 40:399–411. 10.1097/IAE.0000000000002590 31259808

[19] Pan CW Qiu QX Qian DJ Hu DN Li J Saw SM. Iris colour in relation to myopia among Chinese school-aged children. Ophthal Physiol Optics. (2018) 38:48–55. 10.1111/opo.12427 29265474

[20] Shi H Fu J Liu X Wang Y Yong X Jiang L. Influence of the interaction between parental myopia and poor eye habits when reading and writing and poor reading posture on prevalence of myopia in school students in Urumqi, China. BMC Ophthalmol. (2021) 21:299. 10.1186/s12886-021-02058-3 34391397 PMC8364037

[21] Liu S Ye S Xi W Zhang X. Electronic devices and myopic refraction among children aged 6-14 years in urban areas of Tianjin, China. Ophthal Physiol Optics. (2019) 39:282–93. 10.1111/opo.12620 31099434

[22] Xu L Zhuang Y Zhang G Ma Y Yuan J Tu C. Design, methodology, and baseline of whole city-million scale children and adolescents myopia survey (CAMS) in Wenzhou, China. Eye Vis. (2021) 8:31. 10.1186/s40662-021-00255-1 34407890 PMC8373605

[23] Li T Zhou X Zhu J Tang X Gu X. Effect of cycloplegia on the measurement of refractive error in Chinese children. Clin Exp Optomet. (2019) 102:160–5. 10.1111/cxo.12829 30136309 PMC6585953

[24] Zhu D Wang Y Yang X Yang D Guo K Guo Y. Pre- and postcycloplegic refractions in children and adolescents. PLoS ONE. (2016) 11:e0167628. 10.1371/journal.pone.0167628 27907165 PMC5132192

[25] Li Y Xing Y Jia C Ma J Li X Zhou J. Beijing Pinggu childhood eye study: the baseline refractive characteristics in 6- to 12-year-old Chinese primary school students. Front Public Health. (2022) 10:890261. 10.3389/fpubh.2022.890261 35712315 PMC9196872

[26] Chiang SY Weng TH Lin CM Lin SM. Ethnic disparity in prevalence and associated risk factors of myopia in adolescents. J Formos Med Assoc. (2020) 119:134–43. 10.1016/j.jfma.2019.03.004 30910275

[27] Zhang J Li Z Ren J Wang W Dai J Li C. Prevalence of myopia: a large-scale population-based study among children and adolescents in weifang, china. Front Public Health. (2022) 10:924566. 10.3389/fpubh.2022.924566 35958863 PMC9358211

[28] Rai BB Ashby RS French AN Maddess T. Rural-urban differences in myopia prevalence among myopes presenting to Bhutanese retinal clinical services: a 3-year national study. Graefes Arch Clin Exp Ophthalmol. (2021) 259:613–21. 10.1007/s00417-020-04891-6 32803328

[29] Ramamurthy D Lin Chua SY Saw SM A. review of environmental risk factors for myopia during early life, childhood and adolescence. Clin Exp Optomet. (2015) 98:497–506. 10.1111/cxo.12346 26497977

[30] Lee SS Mackey DA. Prevalence and risk factors of myopia in young adults: review of findings from the Raine study. Front Public Health. (2022) 10:861044. 10.3389/fpubh.2022.861044 35570945 PMC9092372

[31] Gupta S Joshi A Saxena H Chatterjee A. Outdoor activity and myopia progression in children: a follow-up study using mixed-effects model. Indian J Ophthalmol. (2021) 69:3446–50. 10.4103/ijo.IJO_3602_20 34826972 PMC8837331

[32] Morgan IG French AN Ashby RS Guo X Ding X He M. The epidemics of myopia: aetiology and prevention. Prog Retin Eye Res. (2018) 62:134–49. 10.1016/j.preteyeres.2017.09.004 28951126

[33] Cai XB Shen SR Chen DF Zhang Q Jin ZB. An overview of myopia genetics. Exp Eye Res. (2019) 188:107778. 10.1016/j.exer.2019.107778 31472110

[34] Lee SS Lingham G Sanfilippo PG Hammond CJ Saw SM Guggenheim JA. Incidence and progression of myopia in early adulthood. JAMA Ophthalmol. (2022) 140:162–9. 10.1001/jamaophthalmol.2021.5067 34989764 PMC8739830

[35] Rudnicka AR Kapetanakis VV Wathern AK Logan NS Gilmartin B Whincup PH. Global variations and time trends in the prevalence of childhood myopia, a systematic review and quantitative meta-analysis: implications for aetiology and early prevention. Br J Ophthalmol. (2016) 100:882–90. 10.1136/bjophthalmol-2015-307724 26802174 PMC4941141

[36] Li Y Liu J Qi P. The increasing prevalence of myopia in junior high school students in the Haidian District of Beijing, China: a 10-year population-based survey. BMC Ophthalmol. (2017) 17:88. 10.1186/s12886-017-0483-6 28606071 PMC5468969

[37] Zhang C Li L Jan C Li X Qu J. Association of school education with eyesight among children and adolescents. JAMA Netw Open. (2022) 5:e229545. 10.1001/jamanetworkopen.2022.9545 35486402 PMC9055461

[38] Williams KM Bertelsen G Cumberland P Wolfram C Verhoeven VJ Anastasopoulos E. Increasing prevalence of myopia in Europe and the impact of education. Ophthalmology. (2015) 122:1489–97. 10.1016/j.ophtha.2015.03.018 25983215 PMC4504030

[39] Nickels S Hopf S Pfeiffer N Schuster AK. Myopia is associated with education: results from NHANES 1999-2008. PLoS ONE. (2019) 14:e0211196. 10.1371/journal.pone.0211196 30695049 PMC6350963

[40] Plotnikov D Williams C Atan D Davies NM Ghorbani Mojarrad N Guggenheim JA. Effect of education on myopia: evidence from the United Kingdom ROSLA 1972 reform. Invest Ophthalmol Vis Sci. (2020) 61:7. 10.1167/iovs.61.11.7 32886096 PMC7476669

[41] Lanca C Saw SM. The association between digital screen time and myopia: a systematic review. Ophthal Physiol Optics. (2020) 40:216–29. 10.1111/opo.12657 31943280

[42] Foreman J Salim AT Praveen A Fonseka D Ting DSW Guang He M. Association between digital smart device use and myopia: a systematic review and meta-analysis. Lancet Digital Health. (2021) 3:e806–18. 10.1016/S2589-7500(21)00135-7 34625399

[43] Iwase A Araie M Tomidokoro A Yamamoto T Shimizu H Kitazawa Y. Prevalence and causes of low vision and blindness in a Japanese adult population: the Tajimi Study. Ophthalmology. (2006) 113:1354–62. 10.1016/j.ophtha.2006.04.022 16877074

[44] Williams K Hammond C. High myopia and its risks. Commun Eye Health. (2019) 32:5–6. PMC6688422 31409941

[45] Qian DJ Zhong H Li J Niu Z Yuan Y Pan CW. Myopia among school students in rural China (Yunnan). Ophthal Physiol Optics. (2016) 36:381–7. 10.1111/opo.12287 26896871

[46] Singh NK James RM Yadav A Kumar R Asthana S Labani S. Prevalence of myopia and associated risk factors in schoolchildren in North India. Optom Vis Sci. (2019) 96:200–5. 10.1097/OPX.0000000000001344 30801501

[47] Wu PC Huang HM Yu HJ Fang PC Chen CT. Epidemiology of myopia. Asia Pac J Ophthalmol. (2016) 5:386–93. 10.1097/APO.0000000000000236 27898441

